# Exosome-mediated delivery of inflammation-responsive *Il-10* mRNA for controlled atherosclerosis treatment

**DOI:** 10.7150/thno.64229

**Published:** 2021-10-25

**Authors:** Te Bu, Zhelong Li, Ying Hou, Wenqi Sun, Rongxin Zhang, Lianbi Zhao, Mengying Wei, Guodong Yang, Lijun Yuan

**Affiliations:** 1 Department of Ultrasound Diagnostics, Tangdu Hospital, Fourth Military Medical University, Xi'an, People's Republic of China.; 2 The State Laboratory of Cancer Biology, Department of Biochemistry and Molecular Biology, Fourth Military Medical University, Xi'an, People's Republic of China.

**Keywords:** Atherosclerosis, exosomes, interleukin-10, inflammation-responsive, internal ribosome entry site

## Abstract

**Rationale:** Tailored inflammation control is badly needed for the treatment of kinds of inflammatory diseases, such as atherosclerosis. IL-10 is a potent anti-inflammatory cytokine, while systemic and repeated delivery could cause detrimental side-effects due to immune repression. In this study, we have developed a nano-system to deliver inflammation-responsive *Il-10* mRNA preferentially into macrophages for tailored inflammation control.

**Methods:** *Il-10* was engineered to harbor a modified HCV-IRES (hepatitis C virus internal ribosome entry site), in which the two miR-122 recognition sites were replaced by two miR-155 recognition sites. The translational responsiveness of the engineered mRNA to miR-155 was tested by Western blot or ELISA. Moreover, the engineered* Il-10* mRNA was passively encapsulated into exosomes by forced expression in donor cells. Therapeutic effects on atherosclerosis and the systemic leaky expression effects *in vivo* of the functionalized exosomes were analyzed in ApoE^-/-^ (Apolipoprotein E-deficient) mice.

**Results:** The engineered IRES-*Il-10* mRNA could be translationally activated in cells when miR-155 was forced expressed or in M1 polarized macrophages with endogenous miR-155 induced. In addition, the engineered IRES-*Il-10* mRNA, when encapsulated into the exosomes, could be efficiently delivered into macrophages and some other cell types in the plaque in ApoE^-/-^ mice. In the recipient cells of the plaque, the encapsulated *Il-10* mRNA was functionally translated into protein, with relatively low leaky in other tissues/organs without obvious inflammation. Consistent with the robust *Il-10* induction in the plaque, exosome-based delivery of the engineered *Il-10* could alleviate the atherosclerosis in ApoE^-/-^ mice.

**Conclusion:** Our study established a potent platform for controlled inflammation control via exosome-based systemic and repeated delivery of engineered *Il-10* mRNA, which could be a promising strategy for atherosclerosis treatment.

## Introduction

Atherosclerosis (AS), the major cause of cardiovascular disease (CVD), is a chronic inflammatory condition characterized by plaque builds up in the walls of the arteries 1-3. Currently, clinical treatments of atherosclerosis mainly include lifestyle interventions 4, lipid-lowering therapies 5 and anti-thrombotic treatments 6. Accumulating evidence suggests that anti-inflammatory therapy opens a new window for atherosclerosis treatment 7. For example, the therapeutic effects and safety of interleukin-1β inhibitor Canakinumab 8, lipase A2 inhibitor 9, and p38MAPK inhibitor Losmapimod 10 are investigated in clinical trial. Notably, these therapeutics mostly depend on frequently systemic injection, with unexpected side-effects unavoidable. Thus, development of novel anti-inflammatory strategy with high delivery specificity, lower off-target effects is badly needed.

Exosomes are extracellular vesicles originating from endosome with a diameter of about 40-160 nm (an average of about 100 nm) and a density of 1.13-1.18 g/mL, carrying a variety of cargoes, such as proteins and nucleic acids, which can regulate pathological or physiological processes and participate in intercellular communication 11. Exosomes are promising as drug delivery carriers because of the good stability in circulation and strong ability to cross a variety of biological barriers 11-13. However, beyond the target tissues/cells, exosomes are taken up by many other cells throughout the body. Exosome tracking analysis has revealed that exosomes are inevitably endocytosed by liver, spleen, bone marrow and other organs enriched with mononuclear phagocyte system 14, 15. In addition to the surface functionalization of exosomes, development of inflammation-responsive pro-drug holds as a feasible choice.

IL-10, is a soluble cytokine produced by macrophages, regulatory T cells and some other cell types. IL-10 plays a major role in limiting inflammation, preventing tissue damage. Therapeutic delivery of IL-10 has been shown to suppress atherosclerosis 16-19. In this study, *Il-10* mRNA was engineered to harbor a modified HCV-IRES (hepatitis C virus-internal ribosome entry site) responsive to miR-155. The engineered* Il-10* mRNA could be efficiently delivered into macrophages in the plaque in ApoE^-/-^ mice when encapsulated into the exosomes. Exosome-based delivery of the engineered *Il-10* mRNA could alleviate the atherosclerosis in ApoE^-/-^ mice while had minimal side-effects. Our study established a potent platform for tailored inflammation control via exosome-based systemic and repeated delivery of engineered *Il-10* mRNA, which could be a promising strategy for atherosclerosis treatment.

## Results

### Engineering of inflammation-responsive* Il-10* mRNA

It has been found that the IRES of HCV is responsible for the specific translation of viral proteins in liver as the high abundance of miR-122 there could change the conformation of the IRES 20, 21. Replacement of the miR-122 recognition sites in the HCV-IRES to sequences recognized by other miRNAs make it possible to construct miRNA-responsive mRNA practical 22. In order to construct an inflammation-responsive mRNA in the context of atherosclerosis, we first wanted to identify an inflammation-associated miRNA. Previous study 23 has confirmed that the content of miR-155 in plaques of patients with AS is significantly increased. Together, the data suggested that miR-155 might be chosen as the miRNA for construction of inflammation-responsive mRNA.

**Figure 1:**
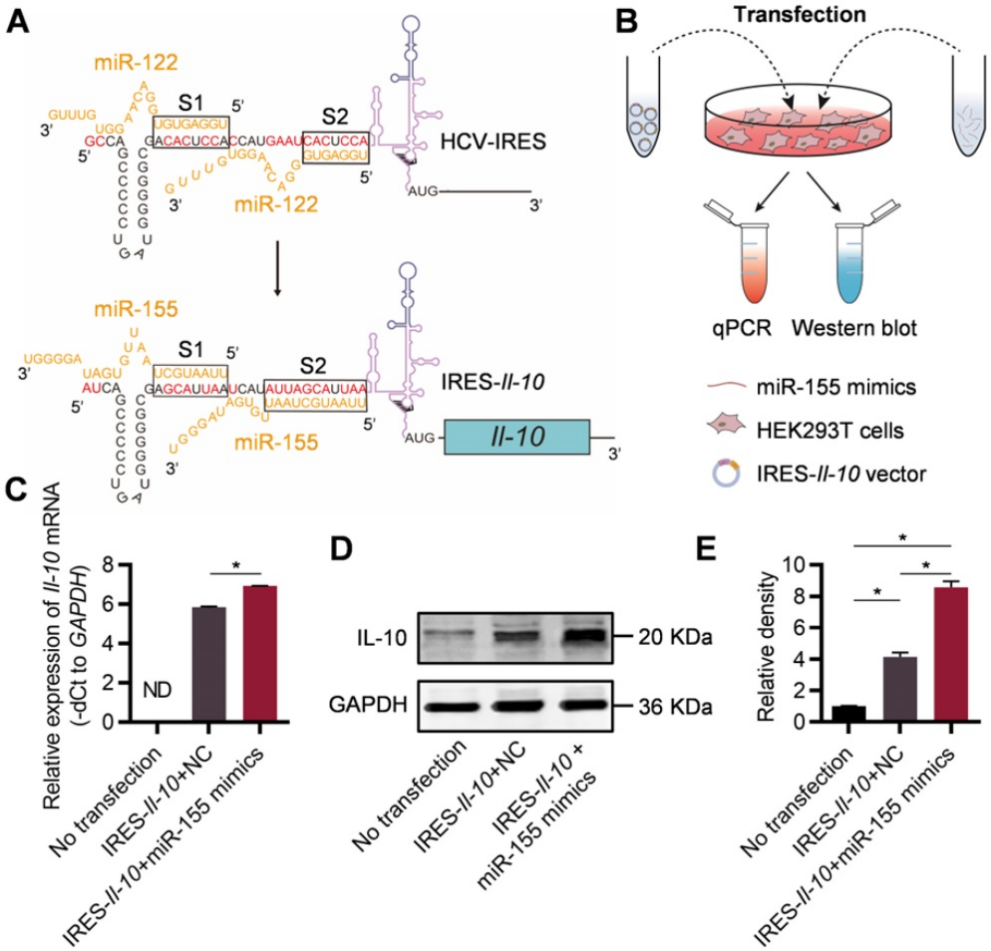
** Engineering of inflammation-responsive *Il-10* mRNA. (A)** Schematic of miR-155-activated IRES-*Il-10* mRNA. Binding to miR-122, HCV-IRES underwent conformational changes that were conducive to translation. The miR-122 recognition region of the HCV-IRES was replaced with a miR-155 recognition sequence (the substituted bases were red) and downstream ligated to the CDS of mouse *Il-10* to achieve miR-155-responsive IL-10 translation activation. **(B)** Schematic of verifying the function of miR-155-activated IRES-*Il-10* mRNA at the cellular level by Western blot and qPCR. **(C)** qPCR analysis of *Il-10* mRNA in cells transfected as indicated. *GAPDH* served as an internal control. **(D)** Representative Western blot image of IL-10 protein expression in HEK293T cells transfected as indicated. GAPDH served as the loading control. **(E)** Quantification of Western blot bands by densitometry. Data are presented as mean ± SEM of three independent experiments. *, *p* < 0.05 by one-way ANOVA. NC, negative control. ND, not determined as Ct value greater than 38.

For construction of miR-155-activated *Il-10* mRNA expression system, the IRES sequence of HCV recognized by miR-122 (Figure S1, denoted as S1 and S2) has been changed to the sequence complementary to miR-155 during the cloning of the expressing plasmid (Figure 1A). Upon transfection, the IRES-*Il-10* transcript was synthesized by the cell (Figure S2). To explore the responsiveness of the designed IRES-*Il-10* mRNA to miR-155, miR-155 mimics or negative control (NC) were co-transfected into HEK293T cells (Figure 1B, Figure S3). Upon miR-155 co-transfection, IRES-*Il-10* mRNA abundance was slightly increased (Figure 1C), while the expression of IL-10 at protein level was significantly induced (Figure 1D, E), suggesting that miR-155 could translationally activate the engineered IRES-*Il-10* mRNA.

### Preparation and characterization of Exo^IRES-*Il-10*^

**Figure 2:**
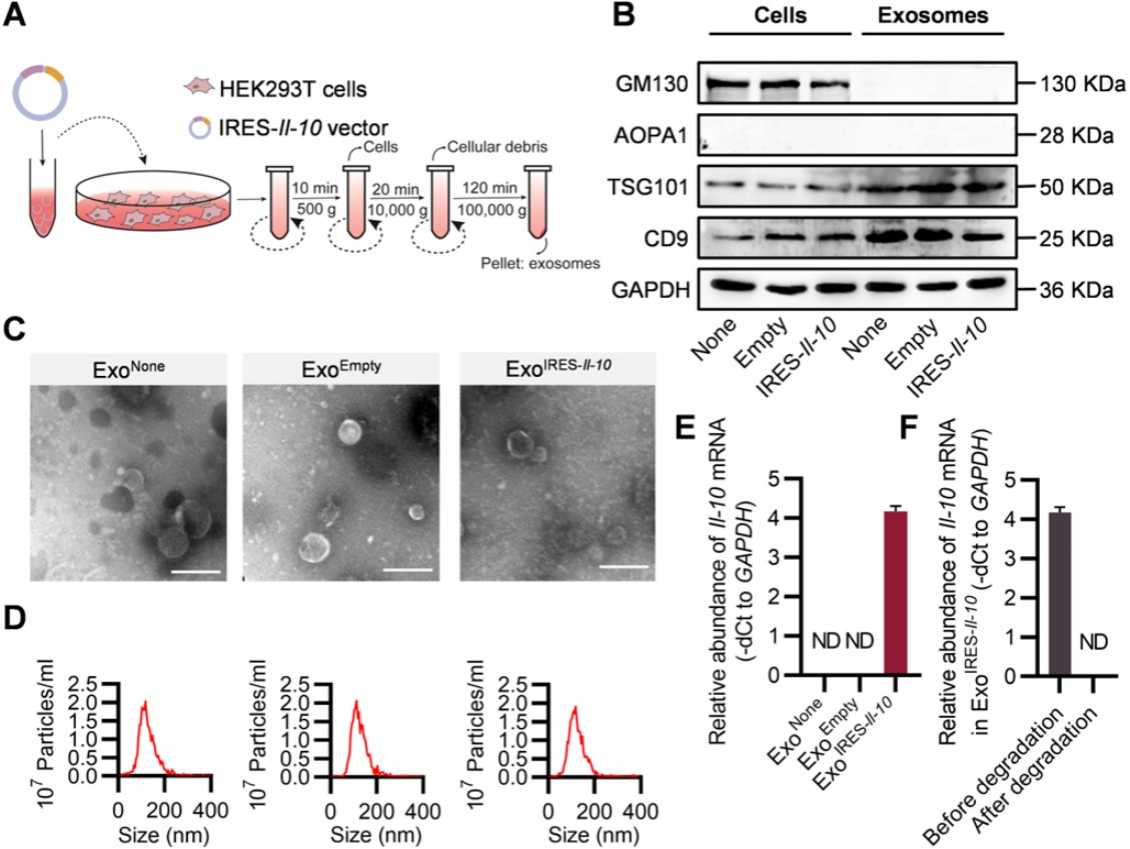
** Preparation and characterization of Exo^IRES-*IL-10*^. (A)** Schematic of the exosomes preparation and isolation process. **(B)** Western blot analysis of the exosomal inclusive markers (TSG101, CD9), exclusive marker (GM130), negative markers to confirm the purity (APOA1) in the isolated exosomes and parental cells. HEK293T cells were treated with PBS or transfected, empty vector, or IRES*-Il-10* plasmids. **(C)** Representative transmission electron microscope (TEM) images of Exo^None^, Exo^Empty^ or Exo^IRES-*IL-10*^. Scale bar = 200 nm. **(D)** Size distribution of the isolated exosomes as indicated. **(E)** qPCR analysis of *Il-10* mRNA in the isolated exosomes as indicated. *GAPDH* served as an internal control. **(F)** qPCR analysis of *Il-10* mRNA in Exo^IRES-*IL-10*^ in exosomal RNA degradation assay as indicated. *GAPDH* served as an internal control. Data are presented as mean ± SEM of three independent experiments. ND, not determined as Ct value greater than 38.

The above data showed that intracellular IRES-*Il-10* mRNA can be translationally activated by inflammation. To deliver IRES-*Il-10* mRNA into the target cells, we chose exosomes as the carrier. Numerous studies used HEK293T cells as donor cells to produce exosomes, as no obvious side-effects were found 24-26. Thus, the IRES-*Il-10* expression plasmids were transfected into the HEK293T cells. The transcribed IRES-*Il-10* mRNA was thus passively loaded into the secreted exosomes, with the exosomes designated as Exo^IRES-*Il-10*^ (Figure 2A). Compared with the control exosomes Exo^None^ (derived from untransfected cells) and Exo^Empty^ (exosomes produced by the cells transfected with empty vector), expression of the exosomal inclusive markers TSG101 and CD9 and exclusive marker GM130 in Exo^IRES*-Il-10*^ had no significant changes. Moreover, there was no expression of APOA1 in exosomes, excluding the serum contaminants (Figure 2B). Transmission electron microscopy (TEM) (Figure 2C) and nanoparticle tracer analysis (NTA) (Figure 2D) further confirmed that Exo^None^, Exo^Empty^ and Exo^IRES-*Il-10*^ all were similar in morphology and size distribution.

To explore whether exosomes derived from HEK293T cells contain oncogenic or other toxic proteins, exosomes were subjected to mass spectrometry analysis. The results showed that the main proteins in the exosomes are not oncogenic or pro-inflammatory (Table S1). Consistently, treatment with the exosomes didn't change the cell viability and proliferation of the recipient cells, though they were efficiently up-taken (Figure S4A-B). Together, our data showed that exosomes derived from HEK-293T had no obvious side-effects, which were consistent with previous studies 24-26.

To test whether transgenic IRES-*Il-10* mRNA has been successfully uploaded into exosomes, we used qPCR to analyze the abundance of *Il-10* mRNA in the isolated exosomes. As expected, IRES-*Il-10* mRNA was robustly encapsulated in Exo^IRES-*Il-10*^, in comparison with the internal control *GAPDH* (Figure 2E). Frankly, there are no standard housekeeping genes for qPCR analysis of exosomal mRNA. To further confirm the findings, U6 was also used as another control. As expected, similar results were observed, though U6 abundance was much higher than *GAPDH* (Figure S5A-C). To exclude the artificial effects of qPCR, we carried out the exosome degradation assay. After degradation of the exosomal RNA, *Il-10* mRNA was not detectable (Figure 2F). Together, these data confirmed that *Il-10* mRNA was efficiently loaded into exosomes.

### Exo^IRES-*Il-10*^delivers miR-155 responsive IRES-*Il-10* mRNA into recipient cells

**Figure 3:**
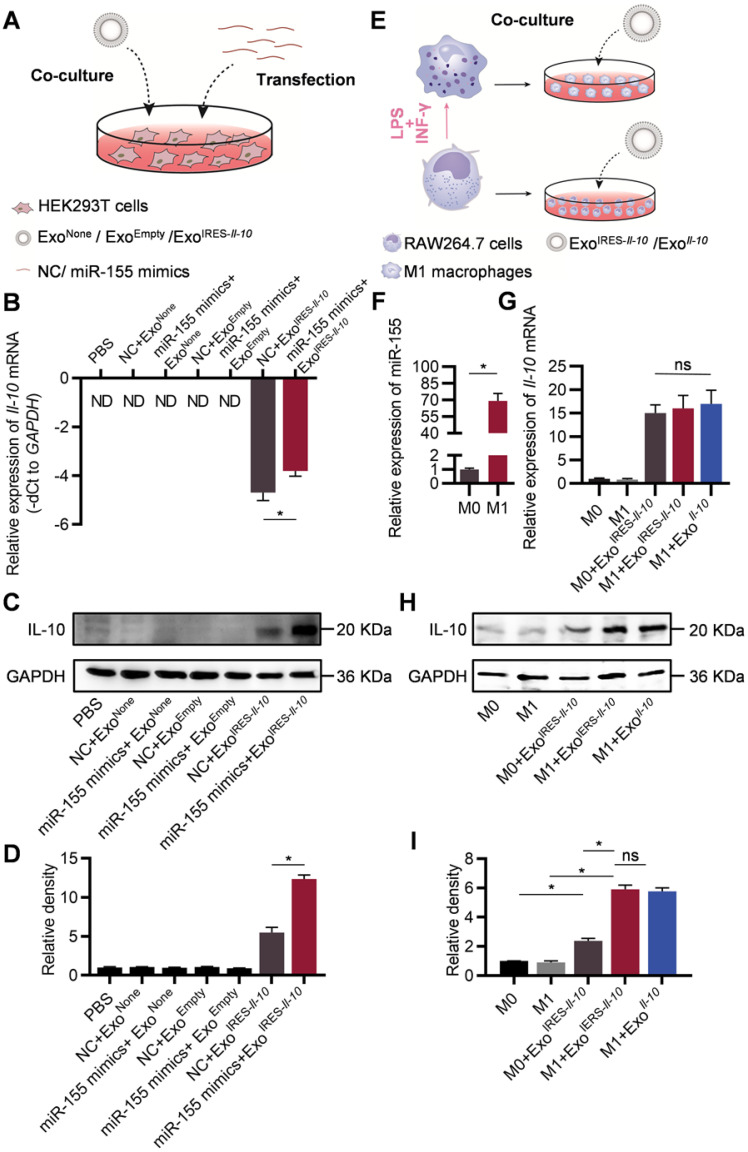
** Engineered IRES-*Il-10* mRNA in Exosomes is translationally activated by miR-155 in recipient cells. (A)** Schematic of exosomes co-cultured with HEK293T cells transfected with miR-155 mimics or NC. **(B)** qPCR analysis of *Il-10* mRNA in HEK293T cells after transfection miR-155 or NC then receiving exosomes as indicated. *GAPDH* served as an internal control. **(C)** Western blot analysis of IL-10 protein expression in HEK293T after transfection miR-155 or NC then receiving exosomes as indicated. GAPDH served as the loading control. **(D)** Quantification of Western blot bands by densitometry. **(E)** Schematic of exosomes co-cultured with polarized macrophages. **(F)** qPCR analysis of miR-155 in M0 macrophages and M1 macrophages. **(G)** qPCR analysis of *Il-10* mRNA in polarized macrophages receiving exosomes as indicated. **(H)** Western blot analysis of IL-10 protein expression in polarized macrophages treated as indicated. GAPDH served as the loading control. **(I)** Quantification of Western blot bands by densitometry. Data are presented as mean ± SEM of three independent experiments. *, *p* < 0.05 by student's *t* test or one-way ANOVA. ns, no significance. ND, not determined as Ct value greater than 38. NC, negative control.

In the following experiments, we explored whether the engineered mRNA delivered by Exo^IRES-*Il-10*^ could be translationally activated by intracellular miR-155. The exosome-treated HEK293T cells were additionally transfected with miR-155 mimics or NC (Figure 3A). qPCR results revealed that miR-155 transfection slightly increased the abundance of IRES-*Il-10* mRNA in Exo^IRES-*Il-10*^ treated HEK293T cells (Figure 3B). However, miR-155 transfection greatly enhanced the expression of IL-10 at protein level in Exo^IRES-*Il-10*^ treated HEK293T cells (Figure 3C, D). We next explored whether endogenous miR-155 in the macrophages could also activate the translation of IRES-*Il-10* mRNA in macrophages. RAW264.7 cells treated with PBS or LPS/IFN-γ were co-cultured with Exo^IRES-*Il-10*^(Figure 3E). Compared with the unpolarized M0, expression of *Il-1β*, *Il-6*, *Tnf-α* and *iNos* in polarized M1 macrophages increased significantly (Figure S6A), while* Il-10* mRNA did not change significantly (Figure S6B). Upon M1 polarization, miR-155 was significantly induced (Figure 3F). Accordingly, IL-10 protein in M1 cells receiving Exo^IRES-*Il-10*^ treatment was robustly enhanced, comparable to the level of Exo*^Il-10^* treatment (encapsulated with capped *Il-10* mRNA without IRES) (Figure 3G-I). These data suggested that Exo^IRES-*Il-10*^ efficiently delivered IRES-*Il-10* mRNA to target cells and the encapsulated IRES-*Il-10* mRNA could be translationally activated by miR-155 in polarized M1 macrophages.

### Exo^IRES-*Il-10*^ alleviates local inflammation in ApoE^-/-^ mice

**Figure 4:**
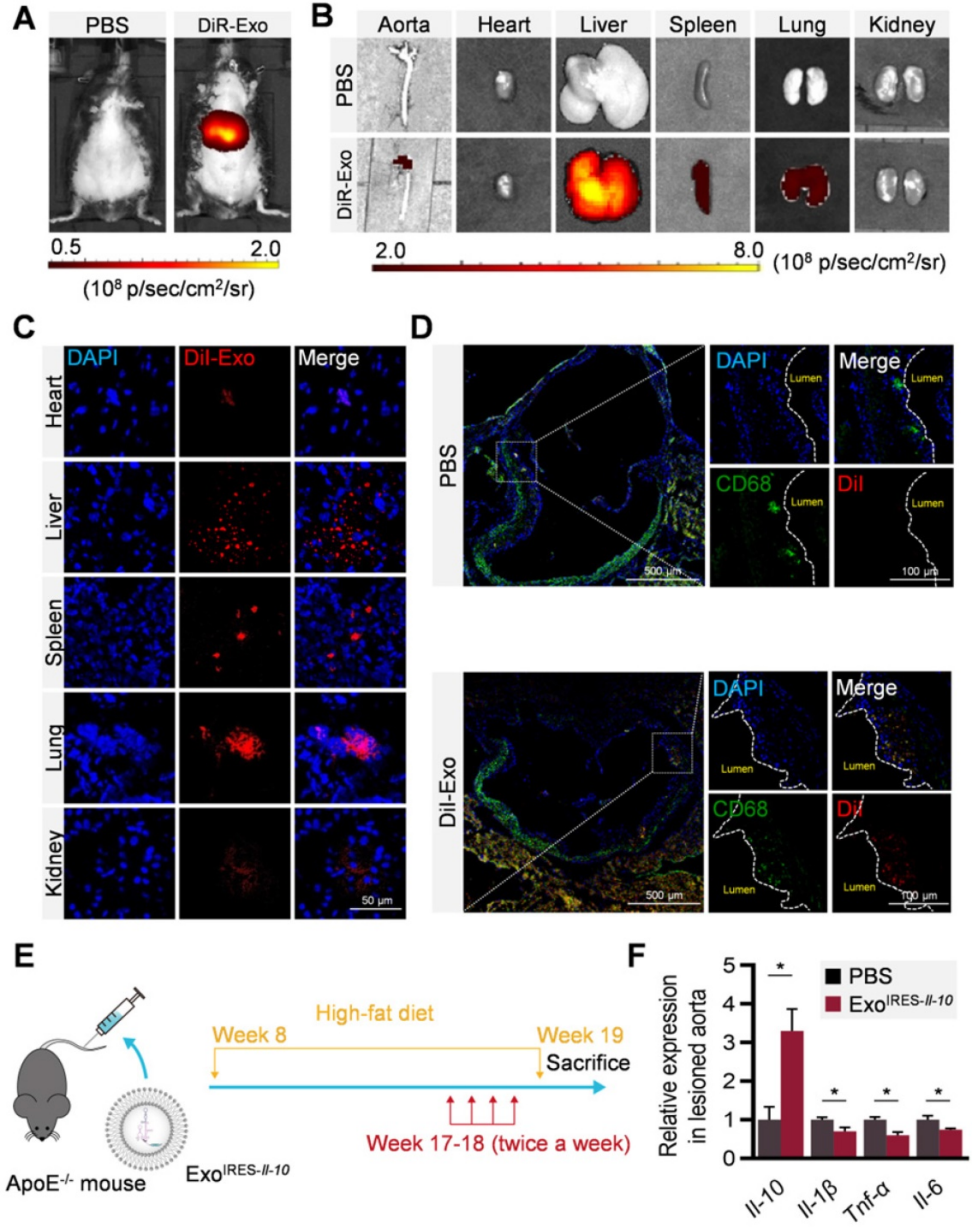
** Local inflammation alleviation by Exo^IRES-*IL-10*^ in ApoE^-/-^ mice. (A)** Representative IVIS images of mice injected with PBS, 200 μg DiR labeled exosomes via tail vein. IVIS imaging was performed 4 h after injection. **(B)** *Ex vivo* fluorescence imaging analysis of the distribution of the DiR-labeled exosomes in different organs, including the aorta, heart, liver, spleen, lung, and kidney. **(C)** Representative confocal images of the localization of DiI-labeled exosomes in different organs. Mice were injected with 200 μg DiI-labeled exosomes via tail vein and sacrificed 4 h after injection. Scale bar = 50 µm. **(D)** Representative confocal images showing the localization of DiI-labeled exosomes in CD68^+^ cells in the atherosclerotic plaques of aortic roots. Scale bar in panorama, 500 µm. Scale bar in magnified image, 100 µm. **(E)** Schematic of the experimental procedure. ApoE^-/-^ mice were fed with a high-fat diet for 8 weeks, followed by the injection of 200 µg Exo^IRES-*IL-10*^ or PBS each time, twice a week, for 2 weeks. Then the mice were sacrificed. **(F)** qPCR analysis of *Il-10* mRNA and inflammation cytokine mRNA levels in lesioned aorta. Data are presented as mean ± SD of three independent experiments. *, *p* < 0.05 by student's *t* test.

To explore whether Exo^IRES-*Il-10*^could deliver the cargos to the plaque, distribution of DiR/DiI-labeled exosomes were tracked by *in vivo* imaging system (IVIS) and microscopic analysis of tissue sections. In accordance with previous findings 27, exosomes were found in all the major organs including the liver, spleen, and lungs (Figure 4A-C). Notably, there were abundant exosomes localized in the plaque in the aorta (Figure 4B). Confocal microscopy further confirmed that the co-localization of exosomes in the CD68^+^ cells (possibly macrophages, macrophage-like smooth muscle cells and some other cell types) in the plaque (Figure 4D). The above data indicated that exosomes entering the plaque could be swallowed by recipient cells in the plaque, and in turn IL-10 protein production was induced if miR-155 were abundantly expressed there. Consistent with the tracing data, two weeks injection of Exo^IRES-*Il-10*^ increased the *Il-10* mRNA while decreased the expression of *Il-1β*, *Tnf-α* and *Il-6* in the plaque (Figure 4E, F). The above results proved that Exo^IRES-*Il-10*^ could deliver functional IRES-*Il-10* mRNA into the plaque for precise control of inflammation.

**Figure 5:**
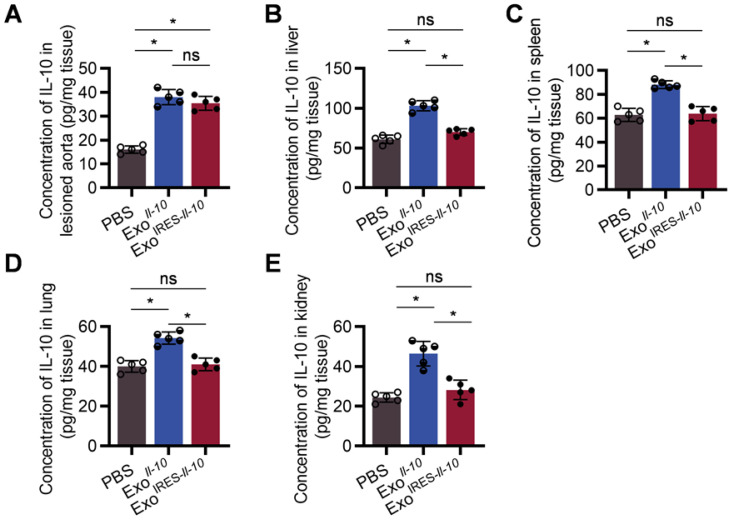
** Exo^IRES-*IL-10*^ precisely induce IL-10 in inflamed tissues in ApoE^-/-^ mice.** ELISA measurement of the concentration of IL-10 protein in lesioned aorta (**A**), liver (**B**), spleen (**C**), lung (**D**), and kidney (**E**) in Apoe^-/-^ mice. Data are presented as mean ± SD. *, *p* < 0.05 by one-way ANOVA. ns, no significance. n = 5 per group.

As shown in Figure 5A, there was no significant difference of IL-10 expression at the protein level between Exo^IRES-*Il-10*^ and Exo*^Il-10^* in the plaque. In contrast, the expression of IL-10 in other organs, such as liver, spleen, lung, kidney, and heart, was much higher in Exo*^Il-10^* group (Figure 5B-E, Figure S7). These results demonstrated that Exo^IRES-*Il-10*^ would have a relatively specific anti-inflammatory effect in the inflammatory sites of atherosclerosis.

### Therapeutic effects of Exo^IRES-*Il-10*^ on atherosclerosis in ApoE^-/-^ mice

**Figure 6:**
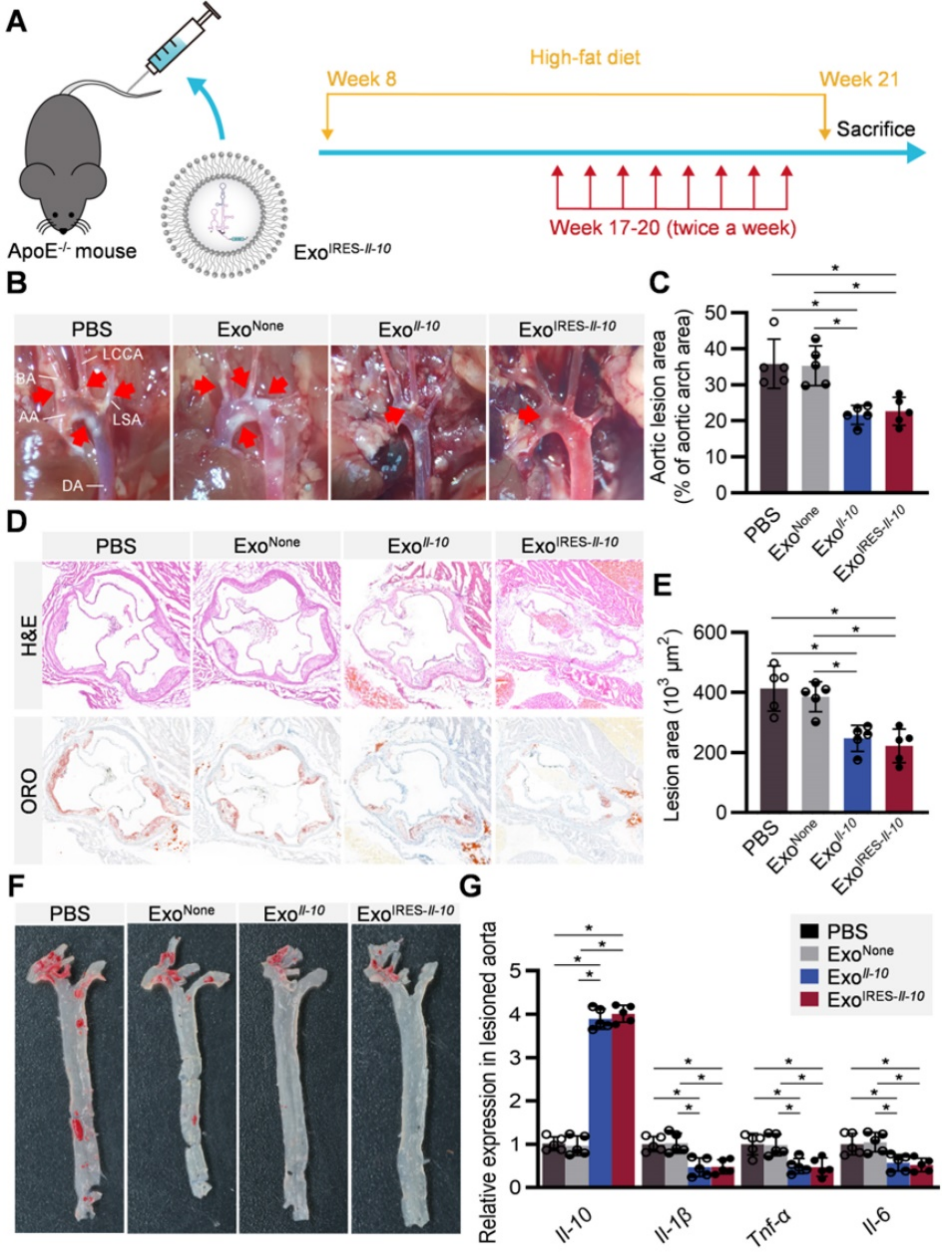
** Therapeutic effects of Exo^IRES-*IL-10*^ on atherosclerotic lesions in ApoE^-/-^ mice. (A)** Schematic of the experimental procedure. ApoE^-/-^ mice were fed with a high-fat diet for 8 weeks, followed by the injection of 200 µg Exo^IRES-*IL-10*^, 200 µg Exo*^IL-10^* or PBS each time, twice a week, for 4 weeks. **(B)** Representative aortic arch view of the atherosclerotic lesions in ApoE^-/-^ mice treated as indicated. AA, ascending aorta; BA, brachiocephalic artery; LCCA, left common carotid artery; LSA, left subclavian artery; DA, descending aorta. **(C)** Percentage of the atherosclerotic area in the aortic arch treated as above. **(D)** Representative images of cross-sectional view of the aortic roots stained with H&E, ORO from ApoE^-/-^ mice treated as indicated. Scale bars, 500 µm. **(E)** Statistical data of the Oil-Red-O (ORO) positive plaque area from D. **(F)** Representative images of ORO staining of the atherogenic lesion areas in mice treated as above. **(G)** qPCR analysis of inflammatory cytokine mRNA levels in lesioned aorta. Data are presented as mean ± SD. *, *p* < 0.05 by one-way ANOVA. n = 5 per group.

All the above data suggested that Exo^IRES-*Il-10*^might have obvious therapeutic effects on AS while have little toxic effects. ApoE^-/-^ mice fed with high-fat diet for 8 weeks were then injected with indicated exosomes twice a week for 4 weeks (Figure 6A). From the gross view of the aorta, Exo^IRES-*Il-10*^ and Exo*^Il-10^* treated mice had fewer atherosclerotic plaques than the control mice receiving PBS or Exo^None^ treatments (Figure 6B, C). Hematoxylin and eosin (H&E) staining and Oil Red O (ORO) staining further showed that Exo^IRES-*Il-10*^ and Exo*^Il-10^* treatment decreased the lesion size, while no obvious differences of the therapeutic effects were observed between Exo^IRES-*Il-10*^ and Exo*^Il-10^* groups (Figure 6D-F). Moreover, there were no significant differences in lipid levels in serum among all the groups (Figure S8). The above data proved that Exo^IRES-*Il-10*^ could achieve on-demand anti-inflammatory effects for atherosclerosis therapy independent of lipid control.

Consistent with previous studies that HEK293 derived exosomes were safe 25, 26, exosome treatment did not cause any obvious damage in those main organs, as detected by H&E staining (Figure S9). In addition, the liver function biomarkers (alanine transaminase and aspartate aminotransferase), kidney function biomarkers (blood urea nitrogen and creatinine) of mice treated with HEK293T derived exosomes with/without further modification were comparable to PBS-treated group (Figure S10). Echocardiography examination further showed that Exo*^IRES-Il-10^* did not cause obvious damage to cardiac structure (Figure S11). Collectively, these data supported the good safety of Exo^IRES-*Il-10*^ *in vivo.* Notably, we here didn't find any advantages of Exo^IRES-*Il-10*^ in safety vs Exo*^Il-10^*, though the advantages exist theoretically. Beyond the limited sensitivity of the examination method, it is also worth further confirming the advantages of Exo^IRES-*Il-10*^upon long time treatment or under infection challenge.

## Discussion

In this study, we first engineered a miR-155-responsive *Il-10* mRNA for specific translation induction in inflamed cells. Then, we delivered the engineered* Il-10* mRNA via exosomes for the treatment of atherosclerosis. In the ApoE^-/-^ mice model, we confirmed that the strategy could efficiently deliver *Il-10* mRNA into inflamed macrophages in relatively high specificity. Moreover, the encapsulated *Il-10* mRNA was translationally activated by miR-155, while the translation was rarely induced in other tissues/organs without obvious inflammation. Consistent with the robust *Il-10* mRNA induction in the plaque, exosome-based delivery of the engineered *Il-10* mRNA could alleviate the atherosclerosis in ApoE^-/-^ mice.

Anti-inflammatory strategies hold great promise for atherosclerosis treatment. Currently, there are multiple candidates are under clinical trial, such as: (1) inhibition of inflammatory factors or adhesion molecule pathways, such as interleukin-1β inhibitor Canakinumab^8^; (2) blocker of arachidonic acid biosynthesis, such as lipase A2 inhibitor^9^; (3) interference of inflammatory signal pathway, such as p38MAPK inhibitor Losmapimod^10^. We here proposed that exosome-based delivery of the IRES-*Il-10* mRNA could alleviate the atherosclerosis in ApoE^-/-^ mice. As atherosclerosis is a chronic disease, long duration of the treatment is needed and strategies with low off-target effects are of great advantage. The advantages of the proposed Exo^IRES-*Il-10*^ are as follows: (1) The exosomes prevent the encapsulated nucleic acids from degradation by the enzymes in the blood 11, 28. Moreover, compared with synthetic nanoparticles, exosomes are of great advantage in less immunogenicity 29, 30. (2) Macrophages are the main inflammatory cells in atherosclerotic plaques 31. Specifically, the polarization of M1 macrophages aggravates the inflammation response in atherosclerotic plaque 32, 33. Our study suggests that the exosome-based delivery strategy could target macrophages in a relatively high specificity. (3) In our study, we delivered *Il-10* mRNA instead of the protein. mRNAs are easier for manipulation than proteins. Moreover, one mRNA could be translated into multiple copies of protein, allowing low need of drug dose. (4) The engineered mRNA could be translationally activated by inflammatory miR-155. Previous studies 23 and our data showed that miR-155 levels were significantly increased in atherosclerotic plaques. After successful delivery, the IRES-*Il-10* mRNA is translated into IL-10 protein only when miR-155 or other analogues are abundant. In the recipient cells with low expression of miR-155, only tracked IL-10 protein could be induced. All of these make on-demand inflammation alleviation possible.

Development of tissue or stimuli responsive drugs are intensively studied recently, such as light response 34, ultrasound response 35, ROS response 36, pH response 37, which can accurately deliver drugs to the target site in a controlled and safe way, thus avoiding the toxic and side effects. Inspired by the specific translation of HCV RNA due to the HCV-IRES 38, the miR-155-responsive mRNA was developed in the study. miR-155 could activate *Il-10* mRNA translation via changing the structure of the IRES from translation inhibition to translation activation, allowing ribosome binding to the RNA. Besides, we cannot rule out possibility that miR-155 enhanced the stability of* Il-10* mRNA. Different from the traditional mechanism that the 2-7 nt from 5' end of miRNA binds the 3' UTR of mRNA, activation of IRES-*Il-10* mRNA by miR-155 might need a longer matched sequence. In other words, the possibility of non-specific activation by miRNAs with similar core sequence is relatively low. Even if the IRES-*Il-10* mRNAs were activated by those similar miRNAs, the end results might be also beneficial for the disease control, as miRNAs share the exact same core sequence with miR-155 would have similar pro-inflammatory function as well. Together, the proposed engineering strategy sets an example for manipulating stimuli responsive mRNA, which could be generalized to multiple RNAs and types of diseases. Future refinement of the mRNA, including modification of 5' -cap, 3'-poly (A) tail, 5'- and 3'-UTRs, codon optimization 22, 39-43, to further improve the specificity and function remain needed.

In the context of atherosclerosis, the host is challenged by low-grade, chronic systemic inflammation. It is thus reasonable to deduce that the slightly elevated miR-155 throughout the body could activate the translation of IRES-*Il-10* mRNA in a modest way in the organs/tissues beyond the plaque, which could be also beneficial for the disease control. In other words, the system could deliver IL-10 on demand by sensing the level of miR-155 *in vivo*.

In atherosclerotic plaques, CD68 may not be a specific marker for macrophages, for example, smooth muscle cells (SMCs) that dedifferentiate into macrophage-like cells also express CD68. In addition, we directly performed qPCR and Elisa analysis of the lesioned aortas, which contained a variety of cells besides macrophages. Further analysis with LCM (laser capture microdissection) to isolate specific regions/cells of interest 44, would strengthen our findings. It is interesting to examine whether the SMCs in the plaque had higher expression of miR-155 and serve as an important target cell for the proposed strategy. As to the manners how exosomes entered the plaques, we preferred the following two possible routes. On one hand, exosomes could circulate into the plaque via the nutrient arteries, especially when angiogenesis is active in the plaque is considered. On the other hand, the vascular endothelium in the lesioned atherosclerotic plaques is incomplete, and the exosomes could also pass through the endothelial barrier to enter the plaque.

Of course, there remains a lot of work to do before clinical translation. Facile isolation and increasing the yield of Exo^IRES-*Il-10*^ remain big challenges. Current isolation methods of exosomes including differential ultracentrifugation, density gradients, precipitation, filtration, size exclusion chromatography, and immune-capture, can only meet the needs of scientific research 45, 46. Some new separation methods, such as tangential flow filtration (TFF) 47, have also been reported to improve the separation efficiency. Strategies to selectively enrich the target cargos, such as the RNA binding protein aided enrichment 48, should be also developed to increase the loading of therapeutics per exosome. It has been also reported that production of exosomes can be boosted by electrical stimulation of donor cells 49. For quality control of the exosomes, especially establishment of the methods and standards for loading efficiency assessment, are urgently needed. For example, previous studies focused on the small RNA (*e.g.,* miRNA) in the exosomes. However, recently multiple studies have found that functional mRNA could be also encapsulated into exosomes, especially for the overexpressed transgenic RNA 22, 48, 50-52. However, how to quantify the mRNA copies per exosomes remains a technical challenge. Currently, there are even no good choice of the internal control genes, though GAPDH and β-actin are usually used 22, 48-51, 53.

In conclusion, the proposed engineered *Il-10* mRNA could be translationally responsive to miR-155, allowing relatively low leaky effects in non-inflamed tissues. Exosome-based delivery of the engineered* Il-10* mRNA could alleviate the atherosclerosis in ApoE^-/-^ mice while had minimal side-effects, allowing long and systemic application in the future. Generally, our study established a potent platform for tailored inflammation control via exosome-based systemic and repeated delivery of engineered *Il-10* mRNA, which could be a promising strategy for atherosclerosis treatment.

## Materials and methods

### Cell Culture

HEK293T cells and RAW264.7 cells cultured in DMEM high glucose medium (HyClone, Logan, USA) with 10% fetal bovine serum (FBS), 1% L-glutamine (Gibco, Carlsbad, USA), and 1% penicillin/streptomycin (HyClone, Logan, USA) otherwise indicated. The cell culture was maintained in a humidified incubator with 5% CO_2_ at 37 °C, with the medium changed every 2 days.

### Plasmid Construction

The designed IRES-*Il-10* consists of modified HCV-IRES and mouse *Il-10* CDS. Briefly, in the designed IRES*-Il-10*, the miR-122 recognition sites in the HCV-IRES were replaced by miR-155 recognition sites and the modified IRES was fused with mouse *Il-10* CDS in the 3' flank. The whole sequence was synthesized in GenScript and subcloned into pWPI with the Pme1 and BstB1 sites. For control, *Il-10* CDS without IRES was also cloned into pWPI*.* The detailed sequences were listed in Table S2.

### Cell Transfection

HEK293T and RAW264.7 were transfected with control or IRES-*Il-10* plasmids together with miRNA mimics using HighGene Transfection reagent (ABclonal Technology, Wuhan, China) according to the manufacturer's protocol. After 6 h of cell transfection, the medium was changed to the complete medium 10% FBS. The detailed sequences of miRNA were listed in Table S3.

### Isolation and Characterization of Exosome

For the isolation of exosomes, cells were cultured with serum free medium for 48 h before supernatant collection. The cultured medium was then centrifuged at 500 g for 10 min to remove cells and then at 10,000 g for 20 min to eliminate the residual cellular debris, followed by ultracentrifugation at 100,000 g for 2 h. The pellet was washed one time and resuspended in sterile PBS and stored at -80 °C for the following experiments.

For examination of morphology, purified exosomes were fixed with 2.5% glutaraldehyde and then dropped onto the copper mesh. After staining with 12% phosphotungstic acid aqueous solution (pH = 6.5), the exosomes were visualized using the JEM-1230 electron microscope (JEOL, Tokyo, Japan) and the images were taken by an armed camera. For nanoparticle tracking analysis, purified exosomes from different sources were analyzed by the NanoSight NS300 (Malvern, Egham, UK).

For mRNA loading efficiency analysis, the supernatants were incubated with 10 μg/mL RNase A for 30 min prior to exosomes isolation, as exosomal RNAs are resistant to biochemical degradation by RNase A. To confirm the PCR specificity, the exosomes were lysed and processed with RNase A, followed by reverse transcription qPCR analysis as described below.

### Cellular Uptake of Exosomes *in vitro*

RAW264.7 cells cultured in confocal dish were incubated with 40 μg/mL (final concentration) DiI-labeled exosomes for 6 h. Cells were fixed in 4% paraformaldehyde for 10 min at room temperature, followed by three times of PBS wash before further staining. Then the nuclei were stained with 4′,6-diamidino-2-phenylindole (DAPI) (Invitrogen, Waltham, USA). Images were obtained by A1R Spectral Confocal Microscope (Nikon, Tokyo, Japan).

### Translational activation of exosomal IRES-Il-10 mRNA in recipient cells

HEK293T cells were transfected with miR-155 mimics or NC (negative control). Six hours after transfection, the exosomes at the final concentration of 40 μg/mL were added into culture medium, and the expression of *Il-10* mRNA was detected by qPCR after co-culture for 12 h.

RAW 264.7 cells were stimulated with 200 ng/mL LPS (L2630, Sigma, St. Louis, USA) and 25 ng/mL IFN-γ (315-05, PeproTech, Cranbury, USA) to promote M1 polarization. Twelve hours after polarization, the exosomes at the final concentration of 40 μg/mL were added into culture medium, and the expression of *Il-10* mRNA was detected by qPCR after co-culture for 12 h. The expression of IL-10 protein was analyzed by Western blot 48 h after co-culture.

### Animal experiments

All animal experiments were performed under protocols approved by the Animal Care and Use Committee of Fourth Military Medical University. Male ApoE^-/-^ mice (7-week-old, 20-22g) were purchased from the Model Animal Research Center of Nanjing University. Mice were maintained under specific pathogen-free conditions with a 12-h light/12-h dark cycle and the temperature kept between 22 °C and 24 °C. After acclimatization for 7 days, mice were fed a high-fat diet (D12492, Research Diet, 45% kcal from fat, 20% kcal from protein, 20% kcal from carbohydrate) for 8 weeks in order to induce atherosclerosis. For treatments, the model mice were then injected with PBS or indicated exosomes (200 μg each time) twice a week via tail vein, for 2 or 4 weeks. At the end of the experiments, the mice were euthanized (200 mg/kg, sodium pentobarbital, *i.p.*). The aortas of mice were removed under stereo microscope and collected. Blood samples and organs of interest were also isolated and processed for further analysis.

### *In vivo* and *ex vivo* Distribution of Exosomes

To assess the distribution of exosomes *in vivo*, the purified exosomes were incubated with DiR (Invitrogen, Waltham, USA) at a final concentration of 8 μM at 37 °C for 30 minutes, and the free DiR was removed by another round of centrifugation. Then mice were injected with 200 μg DiR-labeled exosomes (DiR-Exo) via the tail vein. Four hours after injection, the distribution of exosomes *in vivo and ex vivo* was detected by the IVIS^®^ Lumina II system (PerkinElmer, Waltham, USA) as instructed.

For microscopic analysis of the distribution of exosomes, the exosomes were labeled by DiI (Invitrogen, Waltham, USA) using the method described above before tail vein injection. Four hours after injection, mice were sacrificed and isolated tissues were obtained and mounted in optimum cutting temperature (OCT) compound. Tissue sections were fixed with 4% paraformaldehyde for 10 min and then counterstained with DAPI (Invitrogen, Waltham, USA). In aortas, the internalization of exosomes by CD68^+^ cells was analyzed by immunofluorescent staining in frozen sections (ab197519, Abcam). The whole process was kept from light. The fluorescence signals for the labeled exosomes/cells and the nuclei were visualized by A1R Spectral Confocal Microscope (Nikon, Tokyo, Japan).

### Cell viability assay

RAW264.7 cells were cultured in 6-well plates, then different groups of exosomes (final concentration is 40 μg/mL) or PBS were added to per well. About 24 h after treatments, the cell viability was detected by CCK8 assay as instructed.

### Western blot

Exosomes, or cells were harvested and subjected to cell lysis buffer (Beyotime Biotechnology, China) supplemented with protease inhibitor cocktail (Roche, Basel, Switzerland) at 4 °C for 30 min. The protein content was determined using a BCA protein assay kit (Thermo Fisher Scientific, Waltham, USA). Equal amounts of proteins were then concentrated on 6% SDS-PAGE and separated by 12% SDS-PAGE (120 V for stacking gel and 160 V for separation gel), followed by transferring to nitrocellulose filter membranes with the ice bath. The membranes were blocked with 5% bovine serum albumin (BSA) in TBS-T (tris buffered saline-Tween 20) for 1 h at room temperature and then incubated with primary antibodies overnight at 4 °C. Antibodies used were anti-IL-10 (sc-32815, Santa Cruz), anti-GM130 (11308-1-AP, Proteintech), anti-TSG101 (ab83, Abcam), anti-CD9 (AF5139, Affinity Biosciences), anti-APOA1 (14427-1-AP, Proteintech), anti-GAPDH (60004-1-Ig, Proteintech). After washing three times in TBS-T, the membranes were incubated with anti-rabbit (7074, CST) or anti-mouse (7076, CST) horseradish peroxidase-conjugated secondary antibodies corresponding to the primary antibodies at room temperature for 1h and then visualized using the Amersham^TM^ enhanced chemiluminescence (ECL) Prime western blotting detection reagents (Cytiva, Little Chalfont, UK). Western blot band intensity was quantified by Image J.

### qPCR Analysis

The lesioned aortas and other tissues of interest were processed with TRIzol^®^ reagent (Invitrogen, Waltham, USA) according to the manufacturer's instructions. miRNA and mRNA were extracted and reversely transcribed to cDNA by miRcute Plus miRNA First-Strand cDNA Kit (TIANGEN, Beijing, China) and First Strand cDNA synthesis kit (Genenode, Beijing, China), respectively, following the manufacturer's protocol. qPCR reactions were performed by FastStart Essential DNA Green Master (Roche, Basel, Switzerland). All PCR reactions were run at least in triplicate, and target RNA expression was normalized to U6 or GAPDH levels. Relative expression was calculated by normalizing to the control samples using the 2^-ΔΔCt^ method otherwise indicated. The sequences of PCR primers are provided in Table S4.

### Serum biochemistry

Blood samples were collected after fasting for 8 h. The whole blood samples were kept at room temperature for 2 h or overnight at 4 °C and centrifuged at 4 °C for 4,000 g for 15 min, then the supernatants were stored at -20 °C for detection. Blood lipid level, liver function and renal function parameters were measured by Chemray 800 and Chemray 240 Chemistry analyzer and reagents (Rayto, Shenzhen, China) at Wuhan Servicebio technology CO, LTD.

### Enzyme-linked immunosorbent assays

The aorta and other organs of mice were removed under stereo microscope, and then the lesioned aortas were isolated. The isolated tissues were collected, weighed, and lysed immediately for analysis of IL-10 protein concentrations with the commercial ELISA kit (Proteintech, Wuhan, China). The assay was performed according to the manufacturer's instruction and the relative concentrations were calculated per tissue weight.

### Histology

Tissues, including the aorta, heart, liver, spleen, lung, and kidney, with the surrounding adipose tissue removed, were fixed at 4% paraformaldehyde for 1 h and then the specimens were transferred to PBS containing 30% sucrose overnight. The specimens were embedded in OCT compound and sectioned. Hematoxylin-eosin (H&E) and ORO staining were performed according to the routine protocol. The size of lesions and the area of lipid core were calculated by ImageJ. The histological changes were analyzed from the H&E staining of the main organs by two independent specialists.

### Protein profiling of exosomes by mass spectrometry

Exosomes isolated were subjected to BangFei Bioscience (Beijing, China) for mass spectrometry and subsequent bioinformatics analyses. Each group contained 3 samples. Briefly, total protein of Exo^None^ and Exo^IRES-*Il-10*^ were extracted with RIPA lysis buffer and electrophoresis was done to grossly assess protein quality. Then, sample proteins were digested by trypsin and separated by high performance liquid chromatography (Easy nLC/Ultimate 3000, Thermo Scientific, USA), followed by mass spectrometry analysis (Orbitrap Fusion Lumos, Thermo Scientific, USA). Data were processed using MaxQuant software (version 2.0.1), with peptide false discovery rate (FDR) ≤ 0.01 and protein FDR ≤ 0.01.

### Echocardiography

Mouse hairs from chest to abdomen were first removed using a chemical hair remover. Then the mice were anesthetized with isoflurane (2% induction, 1.2% maintenance) and placed on a temperature-controlled heating pad for normal body temperature maintenance. Animal echocardiography was performed by experienced technicians using Vevo 2100 Imaging System (FUJIFILM VisualSonics, Canada). The heart rate was kept between 400-500 beats per minute during the examination. Two-dimensional short-axis M-mode echocardiography was conducted at the level of the mid-papillary muscle. Parasternal long axis view (PLAX), short axis view and four-chamber view were scanned for multiple cardiac functional parameters. All evaluation parameters were averaged from five cardiac cycles. Investigators were blinded to the identity of animals.

### Statistical Analysis

Data are expressed as mean ± SEM or mean ± SD. Shapiro-Wilk test was used to determine data distribution normality. Student's *t*-test was used for two group comparison, and one-way analysis of variance (ANOVA) with Tukey's posthoc test was used for comparison among 3 or more groups (Graphpad Prism 8.0). P values of < 0.05 indicate statistical significance.

## Supplementary Material

Supplementary figures and tables.
