# Supplementary material for: Exosome-mediated delivery of inflammation-responsive Il-10 mRNA for controlled atherosclerosis treatment
Source: Theranostics. 2021 Oct 25;11(20):9988–10000. doi: 10.7150/thno.64229 (PMC8581418; doi:10.7150/thno.64229)
Supplement: Supplementary file 1 — Supplementary figures and tables. [file thnov11p9988s1.pdf]

Supplementary data

Exosome-mediated delivery of inflammation–responsive *Il-10* mRNA for controlled atherosclerosis treatment

Te Bu[1,#], Zhelong Li[1,#], Ying Hou[1,#], Wenqi Sun[1], Rongxin Zhang[2], Lianbi Zhao[1], Mengying Wei[2], Guodong Yang[2], Lijun Yuan[1,*]

[1]Department of Ultrasound Diagnostics, Tangdu Hospital, Fourth Military Medical University, Xi'an, People's Republic of China

[2]The State Laboratory of Cancer Biology, Department of Biochemistry and Molecular Biology, Fourth Military Medical University, Xi'an, People's Republic of China

# These authors contributed equally to this article.

* Correspondence authors:

Lijun Yuan, Department of Ultrasound Diagnostics, Tangdu Hospital, Fourth Military Medical University, Xinsi Road NO.569th, 710038, Xi'an, China, email: yuanlj@fmmu.edu.cn ; Tel: +862984777471, Fax: +862984777471.

Table S1 Top 200 proteins in Exo^{None} and Exo$^{IRES\text{-}\textit{Il-10}}$ as profiled by mass spectrometry.

Exo^{None}			Exo$^{IRES\text{-}Il\text{-}10}$		
Accession	Gene names	Intensity	Accession	Gene names	Intensity
P62241	RPS8	2.176E+09	P63244	RACK1	2.335E+09
M0R0P7	RPL18A	1.981E+09	P63261	ACTG1	1.767E+09
P40429	RPL13A	1.901E+09	P50990	CCT8	1.567E+09
C9J9K3	RPSA	1.579E+09	P23396	RPS3	1.437E+09
Q5T8U2	RPL7A	1.231E+09	Q5JP53	TUBB	1.383E+09
H0YMV8	RPS27L	1.052E+09	P04264	KRT1	1.256E+09
P62269	RPS18	9.500E+08	A0A2R8Y6J3	RPL5	1.148E+09
A0A087WYF1	LAMA2	9.494E+08	P13645	KRT10	1.125E+09
P63244	RACK1	9.057E+08	B4DUR8	CCT3	1.058E+09
E7EPB3	RPL14	8.802E+08	B7ZAR1	CCT5	1.039E+09
F8VWS0	RPLP0	6.670E+08	P17987	TCP1	1.031E+09
P23396	RPS3	5.396E+08	P08238	HSP90AB1	1.007E+09
A2A3R7	RPS6	5.107E+08	A0A087WVQ6	CLTC	1.005E+09
P04264	KRT1	4.185E+08	C9J9K3	RPSA	1.002E+09
P17987	TCP1	3.576E+08	E9PQD7	RPS2	9.728E+08
Q02878	RPL6	3.382E+08	A0A0U1RQF0	FASN	9.613E+08
E7EX53	RPL15	3.151E+08	G3V1A1	RPL8	8.817E+08
P78371	CCT2	3.135E+08	P62854	RPS26	8.573E+08
P50991	CCT4	2.990E+08	P62805	H4C1	8.324E+08
P62854	RPS26	2.924E+08	P78371	CCT2	8.217E+08
P62913	RPL11	2.822E+08	Q02878	RPL6	7.971E+08
P50990	CCT8	2.821E+08	P61247	RPS3A	7.924E+08
P25786	PSMA1	2.796E+08	A0A087WYV5	SLIT2	7.916E+08
H0Y5B4	RPL36A	2.772E+08	G3V203	RPL18	7.401E+08
K7ERI7	RPL22	2.721E+08	A0A0G2JIW1	HSPA1B	7.234E+08
P62701	RPS4X	2.677E+08	Q99832	CCT7	7.141E+08
H7C1J4	UHRF1BP1	2.657E+08	P13639	EEF2	7.057E+08
G5E9G0	RPL3	2.603E+08	P40227	CCT6A	6.714E+08
A0A0G2JIW1	HSPA1B	2.572E+08	P14618	PKM	6.571E+08
J3QR09	RPL19	2.389E+08	P62701	RPS4X	6.437E+08
P13639	EEF2	2.341E+08	B5MDF5	RAN	6.414E+08
H0YDD8	RPLP2	2.309E+08	P62913	RPL11	6.386E+08
P40227	CCT6A	2.207E+08	P17066	HSPA6	6.284E+08
P36578	RPL4	2.190E+08	P62906	RPL10A	6.274E+08
V9GZ17	TUBA8	2.037E+08	E7EPB3	RPL14	6.248E+08
D6RB09	RPS3A	2.017E+08	P62241	RPS8	6.046E+08
I3L3X7	PSMB6	1.919E+08	P61313	RPL15	5.871E+08
Q5T985	ITIH2	1.889E+08	P11142	HSPA8	5.748E+08

Accession	Gene names	Intensity	Accession	Gene names	Intensity
P17066	HSPA6	1.854E+08	J3KPS3	ALDOA	5.537E+08
D6RAN4	RPL9	1.697E+08	E5RI99	RPL30	5.514E+08
P25398	RPS12	1.693E+08	P55072	VCP	5.444E+08
A0A087WXM6	RPL17	1.682E+08	H0YLP6	RPL28	5.209E+08
A0A2R8Y8A0	RPS24	1.654E+08	Q71U36	TUBA1A	4.895E+08
Q5ST81	TUBB	1.653E+08	P62424	RPL7A	4.883E+08
H0YJM8	PSMB5	1.642E+08	P18124	RPL7	4.760E+08
Q99832	CCT7	1.599E+08	A0A5F9ZHM4	LDHB	4.753E+08
P62081	RPS7	1.503E+08	M0R210	RPS16	4.664E+08
B0QY89	EIF3L	1.468E+08	P62979	RPS27A	4.419E+08
E9PNE6	HSPA8	1.460E+08	B5MCT8	RPS9	4.406E+08
E5RH77	RPS14	1.422E+08	Q08380	LGALS3BP	4.377E+08
P35527	KRT9	1.317E+08	Q08211	DHX9	4.267E+08
H0YN88	RPS17	1.309E+08	F8VQY6	RPLP0	4.228E+08
M0R0F0	RPS5	1.273E+08	P60866	RPS20	4.129E+08
P15880	RPS2	1.262E+08	Q9Y265	RUVBL1	4.056E+08
H0YDT6	EIF3F	1.182E+08	P40429	RPL13A	4.021E+08
H0YLP6	RPL28	1.070E+08	G5EA09	SDCBP	3.852E+08
B4DUR8	CCT3	1.058E+08	A0A087WXM6	RPL17	3.806E+08
P28066	PSMA5	1.025E+08	P02751	FN1	3.743E+08
G3V1A1	RPL8	9.504E+07	P50991	CCT4	3.536E+08
P55072	VCP	9.372E+07	B0QY89	EIF3L	3.447E+08
A0A087WZK9	EIF3H	9.316E+07	E7EUT5	GAPDH	3.329E+08
P49207	RPL34	9.265E+07	O43854	EDIL3	3.287E+08
F8WEQ7	PDCD6IP	9.138E+07	B5MCW2	RPL3	3.234E+08
G5EA09	SDCBP	9.018E+07	P36578	RPL4	3.205E+08
A0A2R8Y6J3	RPL5	8.821E+07	Q14204	DYNC1H1	3.174E+08
P26373	RPL13	8.762E+07	P23526	AHCY	3.143E+08
A6NMY6	ANXA2P2	8.021E+07	P62269	RPS18	3.009E+08
P00338	LDHA	8.001E+07	P35527	KRT9	2.919E+08
E7EVS6	ACTB	7.249E+07	P07814	EPRS1	2.879E+08
Q13347	EIF3I	6.681E+07	M0R3D6	RPL18A	2.840E+08
H7C123	RPL10	6.098E+07	P62280	RPS11	2.709E+08
K7EP36	LGALS3BP	6.073E+07	A0A494C1B8	TPP2	2.572E+08
F8WE98	FLNA	5.927E+07	P05023	ATP1A1	2.555E+08
C9JKP8	PRKD3	5.915E+07	P62263	RPS14	2.443E+08
B4DNK4	PKM	5.784E+07	P07900	HSP90AA1	2.443E+08
P28070	PSMB4	5.716E+07	J3QSB4	RPL13	2.386E+08
E9PIT3	F2	5.399E+07	K7EMV3	H3-3B	2.311E+08
A0A087WVQ6	CLTC	5.339E+07	O43657	TSPAN6	2.299E+08
J3QSA3	UBB	4.600E+07	P05387	RPLP2	2.260E+08
P12532	CKMT1A	4.443E+07	Q9Y3F4	STRAP	2.212E+08

Accession	Gene names	Intensity	Accession	Gene names	Intensity
G3V3I1	PSMA6	4.399E+07	P42677	RPS27	2.183E+08
K7EKH5	ALDOC	3.816E+07	P00338	LDHA	2.163E+08
I3L3W3	METTL16	3.707E+07	I3L3P7	RPS15A	2.125E+08
J3QRI7	RPL26	3.587E+07	P06748	NPM1	2.118E+08
A0A3B3ITT5	RPL29	3.581E+07	P26641	EEF1G	2.050E+08
F8VUA6	RPL18	3.536E+07	P22314	UBA1	1.934E+08
E7EUT5	GAPDH	3.504E+07	P26640	VARS1	1.886E+08
I3L4N6	SLC12A4	3.429E+07	P28074	PSMB5	1.833E+08
H3BT13	SNRPD3	3.297E+07	Q70UQ0	IKBIP	1.832E+08
K7ENG2	U2AF2	2.840E+07	Q15758	SLC1A5	1.796E+08
E9PM31	CD81	2.762E+07	P68371	TUBB4B	1.765E+08
A0A087X208	AGRN	2.700E+07	D6RBL5	ANXA5	1.748E+08
A0A0J9YY17	DNAH10	2.642E+07	P60174	TPI1	1.713E+08
F6UXX1	SYNCRIP	2.606E+07	Q8WUM4	PDCD6IP	1.708E+08
U3KQK0	H2BC15	2.525E+07	P05556	ITGB1	1.664E+08
G3V3W4	PSMA3	2.395E+07	Q96PQ0	SORCS2	1.644E+08
E7ENZ3	CCT5	2.382E+07	F8VNT9	CD63	1.644E+08
X6R6Z1	ILF2	2.332E+07	P53396	ACLY	1.625E+08
A0A590UJZ9	DMBT1	2.257E+07	B5ME19	EIF3CL	1.596E+08
A0A0U1RQF0	FASN	2.203E+07	Q14152	EIF3A	1.590E+08
Q6UWF7	NXPE4	2.068E+07	E9PJK1	CD81	1.555E+08
J3QL05	SRSF2	2.037E+07	J3QRI7	RPL26	1.554E+08
A0A087WYV6	TSPAN6	1.896E+07	Q9Y230	RUVBL2	1.553E+08
A0A0A0MRX1	ELAVL2	1.891E+07	P49207	RPL34	1.541E+08
Q9BY21	GPR87	1.770E+07	A0A0A0MSI0	PRDX1	1.539E+08
J3KN59	BNIP2	1.765E+07	P10809	HSPD1	1.496E+08
E9PQR3	FTH1	1.701E+07	F8VPD4	CAD	1.494E+08
Q9BYK2	RPS21	1.579E+07	P62258	YWHAE	1.484E+08
M0R1H6	RPS11	1.565E+07	A0A2R8Y8A0	RPS24	1.461E+08
P26641	EEF1G	1.561E+07	P78527	PRKDC	1.440E+08
A0A087X0W0	RAN	1.557E+07	P55884	EIF3B	1.381E+08
A6NHK2	SNRPE	1.520E+07	P60842	EIF4A1	1.348E+08
P0DP25	CALM3	1.331E+07	P25788	PSMA3	1.346E+08
Q8NC51	SERBP1	1.196E+07	P25398	RPS12	1.339E+08
P15104	GLUL	1.057E+07	Q13200	PSMD2	1.275E+08
E9PPJ5	MDK	9.864E+06	A6NMY6	ANXA2P2	1.235E+08
A0A2H2FF08	ZFYVE26	9.367E+06	P62753	RPS6	1.233E+08
H3BNU8	KNOP1	9.278E+06	P25705	ATP5F1A	1.230E+08
M0QXF2	ATP1A3	8.308E+06	M0R0F0	RPS5	1.226E+08
E9PJD9	RPL27A	8.210E+06	A0A087WZK9	EIF3H	1.220E+08
E7EUH7	PUS7	7.969E+06	P28066	PSMA5	1.208E+08
E9PFK9	RABGEF1	7.917E+06	A6NNI4	CD9	1.190E+08

Accession	Gene names	Intensity	Accession	Gene names	Intensity
P10599	TXN	7.863E+06	Q9UMS4	PRPF19	1.166E+08
A0A1B0GVI0	SASH1	7.765E+06	P04114	APOB	1.159E+08
E7EX17	EIF4B	7.485E+06	Q969P0	IGSF8	1.157E+08
J3QSX4	BUB3	7.474E+06	P29966	MARCKS	1.139E+08
F8VV56	CD63	7.200E+06	J3QQM1	PSMC5	1.114E+08
C9J6S2	ATP1B3	7.169E+06	C9K025	RPL35A	1.091E+08
A6NG31	PLAAT4	7.133E+06	Q13347	EIF3I	1.073E+08
A0A140T907	FLOT1	7.117E+06	M0R1Y2	KDELR1	1.070E+08
H3BQ38	IST1	6.980E+06	E9PPJ5	MDK	1.036E+08
Q86XP3	DDX42	6.429E+06	P50995	ANXA11	1.018E+08
H0YDZ5	HIF3A	6.306E+06	Q9P2B2	PTGFRN	1.010E+08
Q9UL25	RAB21	6.288E+06	Q96PC5	MIA2	9.714E+07
A0A087WYV5	SLIT2	6.163E+06	Q9Y3U8	RPL36	9.654E+07
Q5TBG5	PSMB7	6.072E+06	E7EX29	YWHAZ	9.499E+07
A0A087WT48	DNAJA2	5.885E+06	A0A2R8Y6G6	ENO1	9.398E+07
F2Z3C0	RPS9	5.596E+06	E9PRK8	FTH1	9.330E+07
A0A2R8YEM2	CCNH	5.485E+06	P81605	DCD	9.307E+07
P50995	ANXA11	5.446E+06	O43684	BUB3	9.224E+07
K7ES31	EIF3K	5.429E+06	P19338	NCL	9.152E+07
H0YA55	ALB	5.288E+06	A0A087WUV8	BSG	9.098E+07
B4E171	CD9	5.217E+06	E9PK47	PYGL	9.036E+07
A0A024RA52	PSMA2	5.102E+06	A0A087WVQ9	EEF1A1	8.946E+07
A0A5F9ZHA0	NF2	4.814E+06	P49006	MARCKSL1	8.845E+07
Q01650	SLC7A5	4.778E+06	F8WF69	CLTA	8.779E+07
Q5T5C7	SARS1	4.663E+06	P35579	MYH9	8.775E+07
E9PF17	VCAN	4.605E+06	Q5QNW6	H2BC18	8.565E+07
P51148	RAB5C	4.435E+06	O14744	PRMT5	8.273E+07
C9IYW8	SLCO1A2	4.355E+06	D3YTB1	RPL32	8.152E+07
Q5SX91	GDI2	4.343E+06	E9PP50	CFL1	8.144E+07
C9J673	XPO1	4.213E+06	P61163	ACTR1A	8.016E+07
Q5T6W2	HNRNPK	3.913E+06	P62851	RPS25	7.880E+07
P78527	PRKDC	3.791E+06	P22234	PAICS	7.766E+07
P55884	EIF3B	3.747E+06	E9PJD9	RPL27A	7.737E+07
Q9UHI8	ADAMTS1	3.651E+06	P04181	OAT	7.515E+07
A0A0A0MRJ9	ARHGAP30	3.613E+06	H7BZ35	DARS1	7.483E+07
Q08945	SSRP1	3.543E+06	P32969	RPL9	7.094E+07
Q9UP65	PLA2G4C	3.495E+06	P51665	PSMD7	6.983E+07
P11047	LAMC1	3.466E+06	P63267	ACTG2	6.947E+07
G3V507	PRMT5	3.290E+06	O00232	PSMD12	6.745E+07
F5H6D6	UBE3B	3.284E+06	B3KVK2	GNB1	6.733E+07
Q9UN81	L1RE1	3.200E+06	Q08431	MFGE8	6.726E+07
A0A2R8YEM9	TRANK1	3.168E+06	P17302	GJA1	6.605E+07

Accession	Gene names	Intensity	Accession	Gene names	Intensity
P29966	MARCKS	3.067E+06	Q16576	RBBP7	6.447E+07
A0A669KBC4	DNAAF6	3.060E+06	H7C3M9	FAM228A	6.353E+07
H0YAW4	EIF3E	3.041E+06	H0YLS6	PSMA4	6.234E+07
M0QXM4	SLC1A5	3.022E+06	P10909	CLU	6.049E+07
J3KRE4	NCOR1	2.937E+06	P62191	PSMC1	6.020E+07
U3KPX5	ITGBL1	2.865E+06	S4R456	RPS15	5.938E+07
A0A1B0GTR9	JAK2	2.838E+06	O00303	EIF3F	5.850E+07
E9PBS1	PAICS	2.829E+06	E9PKG1	PRMT1	5.848E+07
J3QQM1	PSMC5	2.785E+06	Q15170	TCEAL1	5.846E+07
G5E9W8	GYG1	2.657E+06	A0A286YF34	PHGDH	5.818E+07
E5RGE1	YWHAZ	2.537E+06	A0A140T9R1	FLOT1	5.802E+07
A0A494C1B8	TPP2	2.484E+06	F5H5D3	TUBA1C	5.778E+07
D6R9D0	NPY1R	2.452E+06	A0A087WWY3	FLNA	5.765E+07
A0A087X0B4	ANK3	2.395E+06	Q4VCS5	AMOT	5.727E+07
H0Y449	YBX1	2.357E+06	P02786	TFRC	5.631E+07
O15230	LAMA5	2.266E+06	F8W7C6	RPL10	5.622E+07
F2Z329	TMEM237	2.263E+06	E7EX90	DCTN1	5.614E+07
H3BLV9	SRPK1	2.229E+06	H3BQF7	IST1	5.508E+07
A0A3B3ISK1	COPA	2.014E+06	P47897	QARS1	5.486E+07
P13521	SCG2	1.952E+06	Q15008	PSMD6	5.467E+07
P85037	FOXK1	1.942E+06	P67809	YBX1	5.466E+07
E5RHQ2	PPP2R2A	1.937E+06	Q53EZ4	CEP55	5.451E+07
A0A5K1VW66	NF2	1.927E+06	P27348	YWHAQ	5.387E+07
P49006	MARCKSL1	1.900E+06	Q5T5C7	SARS1	5.355E+07
H0Y5I7	SFI1	1.889E+06	P61221	ABCE1	5.353E+07
H0YJJ8	EIF2B2	1.831E+06	P32971	TNFSF8	5.301E+07
Q9H788	SH2D4A	1.776E+06	G3V3I1	PSMA6	5.248E+07
Q7Z2Y8	GVINP1	1.708E+06	J3KSR8	SRSF1	5.201E+07
A6NKB8	RNPEP	1.492E+06	P49189	ALDH9A1	5.193E+07
Q7KZF4	SND1	1.491E+06	P12277	CKB	5.161E+07
A0A087WXQ8	PSMB3	1.471E+06	C9J1Z8	ARF5	5.103E+07
P56545	CTBP2	1.421E+06	Q01650	SLC7A5	5.097E+07
P62877	RBX1	1.412E+06	P13611	VCAN	5.083E+07
B8ZZQ6	PTMA	1.344E+06	O96019	ACTL6A	4.880E+07
V9GYU2	ANKRD54	1.256E+06	B4DY09	ILF2	4.805E+07
Q6PKG0	LARP1	1.222E+06	P11021	HSPA5	4.679E+07

Table S2 mRNA sequences used in the study.

**Sequence of HCV-IRES**
GCCAGCCCCCUGAUGGGGGCGACACUCCACCAUAGAUCACUCCCCUGUGAGGAACUACUGUCUUCACGCAGAAAGCGUCUAGCCAUGGCGUUAGUAUGAGUGUCGUGCAGCCUCCAGGACCCCCCCUCCCGGGAGAGCCAUAGUGGUCUGCGGAACCGGUGAGUACACCGGAAUUGCCAGGACGACCGGGUCCUUUCUUGGAUCAACCCGCUCAAUGCCUGGAGAUUUGGGCGUGCCCCCGCGAGACUGCUAGCCGAGUAGUGUUGGGUCGCGAAAGGCCUUGUGGUACUGCCUGAUAGGGUGCUUGCGAGUGCCCCGGGAGGUCUCGUAGACCGUGCACCAUGAGCACGAAU
**Sequence of miR-155 responsive IRES**
AUCAGCCCCCUGAUGGGGGCGAGCAUUAAUCAUAUUAGCAUUACCUGUGAGGAACUACUGUCUUCACGCAGAAAGCGUCUAGCCAUGGCGUUAGUAUGACAGUCGUGCAGCCUCCAGGACCCCCCCUCCCGGGAGAGCCAUAGUGGUCUGCGGAACCGGUGAGUACACCGGAAUUGCCAGGACGACCGGGUCCUUUCUUGGAUCAACCCGCUCAAUGCCUGGAGAUUUGGGCGUGCCCCCGCGAGACUGCUAGCCGAGUAGUGUUGGGUCGCGAAAGGCCUUGUGGUACUGCCUGAUAGGGUGCUUGCGAGUGCCCCGGGAGGUCUCGUAGACCGUGCACCAUGAGCACGAAU
**Sequence of mouse *Il-10* CDS**
ATGCCTGGCTCAGCACTGCTATGCTGCCTGCTCTTACTGACTGGCATGAGGATCAGCAGGGGCCAGTACAGCCGGGAAGACAATAACTGCACCCACTTCCCAGTCGGCCAGAGCCACATGCTCCTAGAGCTGCGGACGGCCTTCAGCCAGGTGAAGACGTTCTTTCAAACAAAGGACCAGCTGGACAACATACTGCTAACCGACTCCTTAATGCAGGATTTTAAGGGTTACTTGGGTTGCCAAGCCTTATCGGAAATGATCCAGTTTTACCTGGTAGAAGTGATGCCCCAGGCAGAGAAGCATGGCCCAGAAATCAAGGAGCATTTGAATTCCCTGGGTGAGAAGCTGAAGACCCTCAGGATGCGGCTGAGGCGCTGTCATCGATTTCTCCCCTGTGAAAATAAGAGCAAGGCAGTGGAGCAGGTGAAGAGTGATTTTAATAAGCTCCAAGACCAAGGTGTCTACAAGGCCATGAATGAATTTGACATCTTCATCAACTGCATAGAAGCATACATGATGATCAAAATGAAAAGCTAA

Table S3 miRNA sequences used in the study.

miRNA	Sense	Antisense
Negative control	5'UUCUCCGAACGUGUCACGUTT3'	5'ACGUGACACGUUCGGAGAATT 3'
miR-155 mimics	5'UUAAUGCUAAUUGUGAUAGGGG3'	5'CCUAUCACAAUUAGCAUUAAUU3'

Table S4 Primers used in the study.

Primer	Forward	Reverse
mouse *Il-10*	5'GCTCTTACTGACTGGCATGAG3'	5'CGCAGCTCTAGGAGCATGTG3'
mouse *Tnf-α*	5'CTGAACTTCGGGGTGATCGG3'	5'GGCTTGTCACTCGAATTTTGAGA3'
mouse *Arg-1*	5'CTCCAAGCCAAAGTCCTTAGAG3'	5'AGGAGCTGTCATTAGGGACATC3'
mouse *Mrc1*	5'CTCTGTTCAGCTATTGGACGC3'	5'CGGAATTTCTGGGATTCAGCTTC3'
mouse *Fizz1*	5'CCAATCCAGCTAACTATCCCTCC3'	5'ACCCAGTAGCAGTCATCCCA3'
mouse *Il-1β*	5'GCAACTGTTCCTGAACTCAACT3'	5'ATCTTTTGGGGTCCGTCAACT3'
mouse *iNos*	5'GTTCTCAGCCCAACAATACAAGA3'	5'GTGGACGGGTCGATGTCAC3'
mouse *Il-6*	5'TAGTCCTTCCTACCCCAATTTCC3'	5'TTGGTCCTTAGCCACTCCTTC3'
mouse *Gapdh*	5'AGGTCGGTGTGAACGGATTTG3'	5'GGGGTCGTTGATGGCAACA3'
human *GAPDH*	5' CAATGACCCCTTCATTGACC3'	5' GACAAGCTTCCCGTTCTCAG3'
miR-155	5'TTAATGCTAATTGTGATAGGGGT3'	Provided in the Kit
U6	5'CTCGCTTCGGCAGCACA3'	5'AACGCTTCACGAATTTGCGT3'

Supplementary Figures and Figure legends

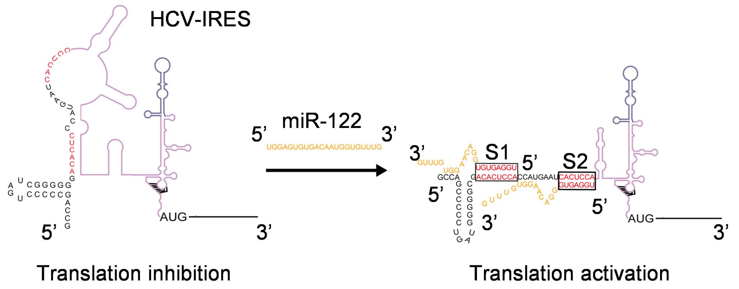

Figure S1 Theoretical conformational change of the HCV-IRES structure in the presence of miR-122. The two sequences of HCV IRES recognized by miR-122 are denoted as S1 and S2 respectively.

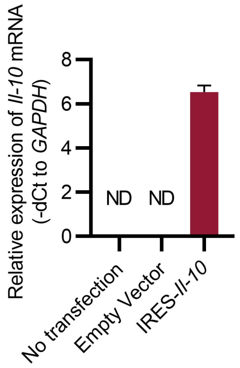

Figure S2 Transfection efficiency of *Il-10* in HEK293T cells. qPCR analysis of *Il-10* mRNA in HEK293T cells treated as indicated. *GAPDH* served as an internal control. Data are expressed as mean ± SEM of three independent experiments. ND, not determined as Ct value greater than 38.

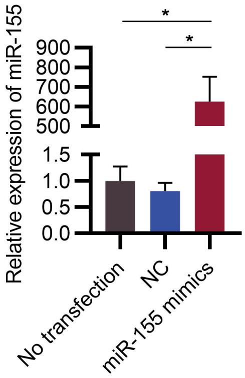

Figure S3 Transfection efficiency of miR-155 in HEK293T cells. qPCR analysis of miR-155 in HEK293T cells treated as indicated. *U6* served as an internal control. Data are expressed as mean ± SEM of three independent experiments. *, $p < 0.05$ by one-way ANOVA. NC, negative control.

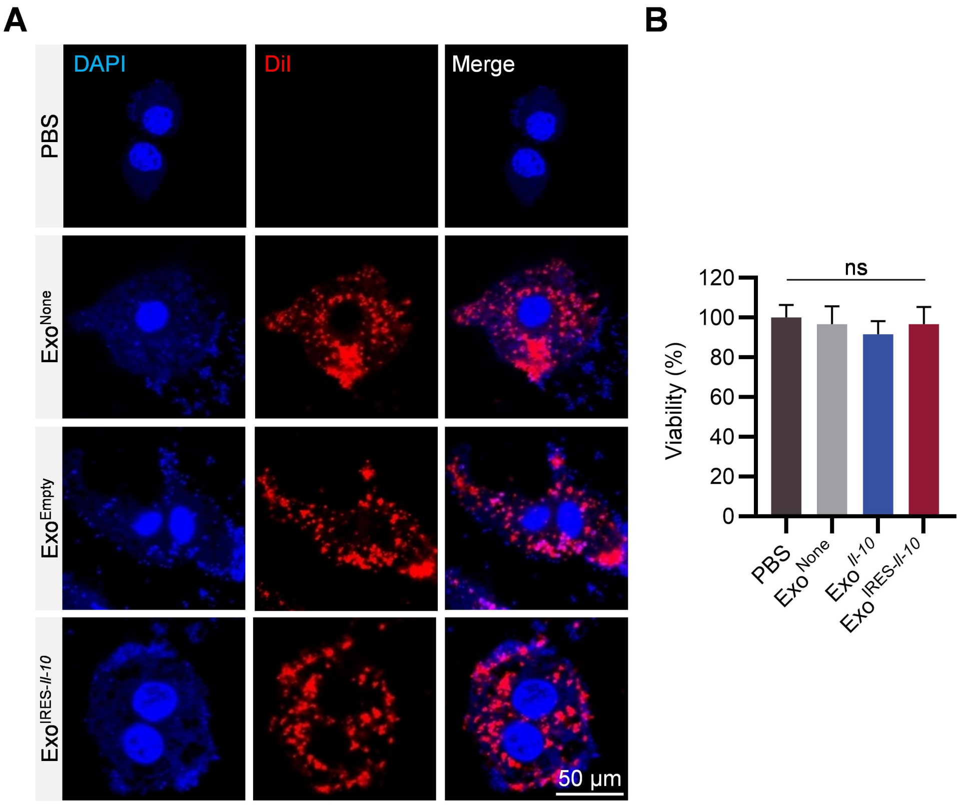

Figure S4 Toxicity of HEK293T derived exosomes on macrophages. (A) Uptake of exosomes in macrophages. RAW264.7 cells were treated with 40 μg/mL (final concentration) DiI-labeled exosomes as indicated for 6 hours. Representative confocal images of DiI-labeled exosomes taken up by cells. Nuclei were counterstained with DAPI. PBS served as negative control. Scale bar = 50 μm. (B) CCK8 assay the viability of RAW264.7 cells that were treated with Exo^{None}, Exo^{Empty} or $Exo^{IRES\text{-}Il\text{-}10}$ (final concentration is 40 μg/mL) or PBS for 24h. Data are expressed as the mean ± SEM of three independent experiments. Data analyzed by one-way ANOVA. ns, no significant difference.

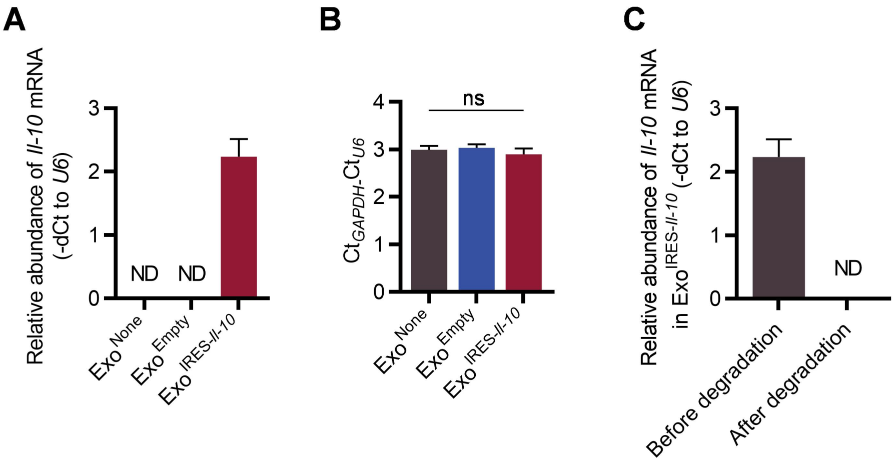

Figure S5 Loading efficiency of IRES-*Il-10* mRNA in exosomes. (A) qPCR analysis of *Il-10* mRNA abundance in the isolated exosomes as indicated. *U6* served as an internal control. (B) Values of Ct_{GAPDH} -Ct_{U6} among different exosomes. (C) qPCR analysis of *ll-10* mRNA in $Exo^{IRES\text{-}Il\text{-}10}$ in exosomes before and after degradation processing. *U6* served as an internal control. Data are expressed as mean ± SEM of three independent experiments. Data are analyzed by one-way ANOVA. ND, not determined as Ct value greater than 38. ns, no significant difference.

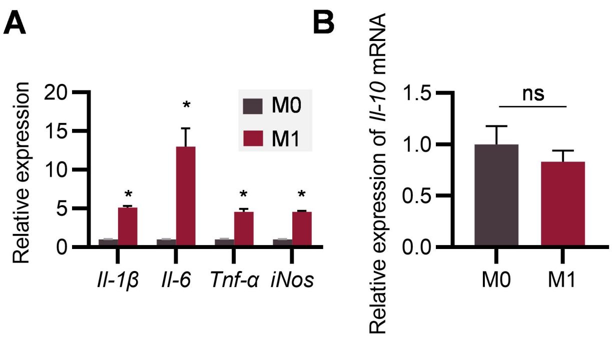

Figure S6 Changes of gene expression in macrophages polarized to M1. (A) qPCR analysis of inflammatory cytokine expression in M0 and M1 macrophages. (B) qPCR analysis of *Il-10* mRNA abundance in M0 and M1 macrophages. Data are expressed as mean ± SEM of three different experiments. *, $p < 0.05$ by student's *t* test. ns, no significance.

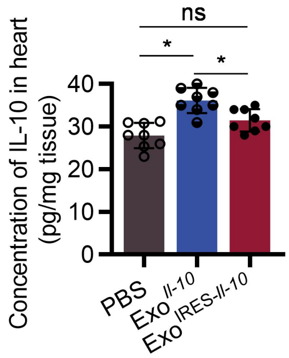

Figure S7 Exo$^{\text{IRES-}\textit{Il-10}}$ rarely induces IL-10 expression in heart in ApoE$^{-/-}$ mice. ELISA measurement of the concentration of IL-10 protein heart in ApoE$^{-/-}$ mice. Data are present as mean ± SD. *, $p < 0.05$ (one-way ANOVA). ns, no significance. n = 8 per group.

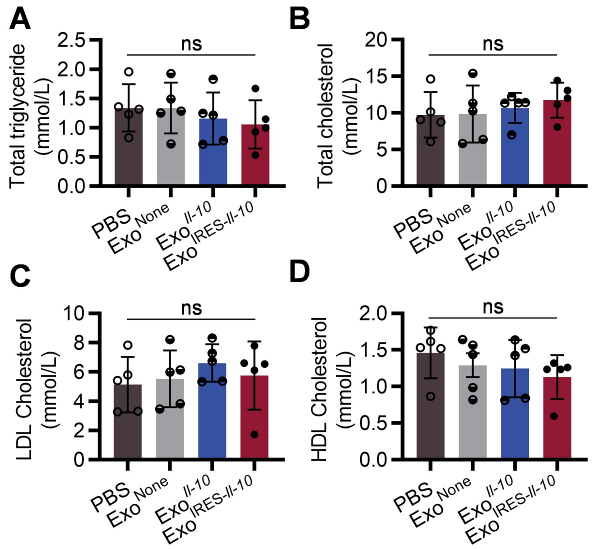

Figure S8 Blood lipid level change in mice receiving different exosome treatments. Examination of the total triglyceride (A), total cholesterol (B), LDL-Cholesterol (C), and HDL-Cholesterol (D) in ApoE$^{-/-}$ mice treated as indicated. Data are expressed as mean ± SD. Data analyzed by one-way ANOVA. ns, no significant difference. n = 5 per group. LDL, low-density lipoprotein; HDL, high-density lipoprotein.

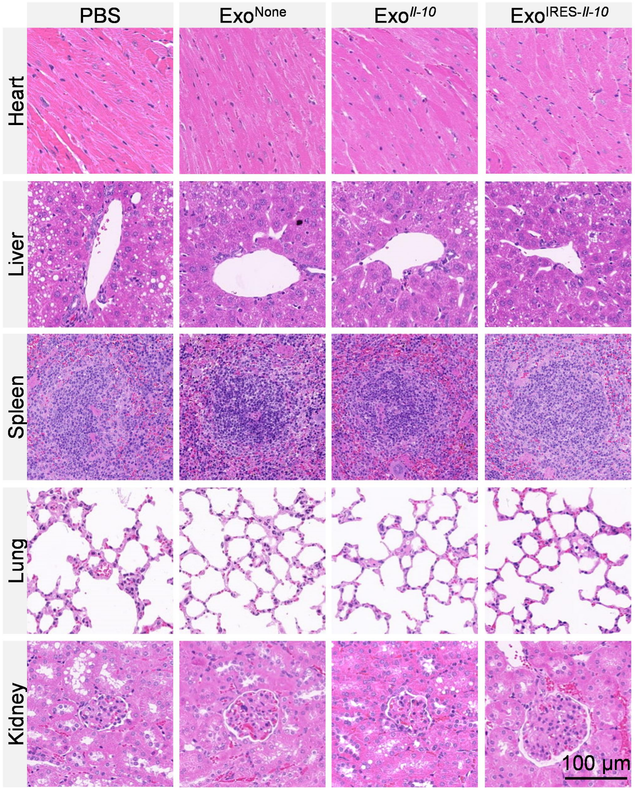

Figure S9 Histological analysis of main organs from mice receiving different exosome treatments. H&E staining of different organs from mice with indicated groups. No significant damage was observed in the heart, liver, spleen, lung, and kidney from histological studies. Scale bars, 100 μm.

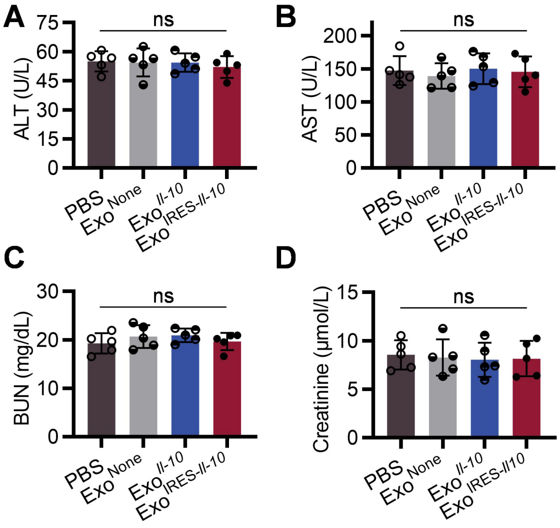

Figure S10 Changes of liver and kidney function in mice receiving different exosome treatments. Examination of the ALT (A), AST (B), BUN (C), and Creatinine (D) in ApoE$^{-/-}$ mice treated as indicated. Data are expressed as mean ± SD. Data are analyzed by one-way ANOVA. ns, no significant difference. n = 5 per group. ALT, alanine aminotransferase; AST, aspartate aminotransferase; BUN, blood urea nitrogen.

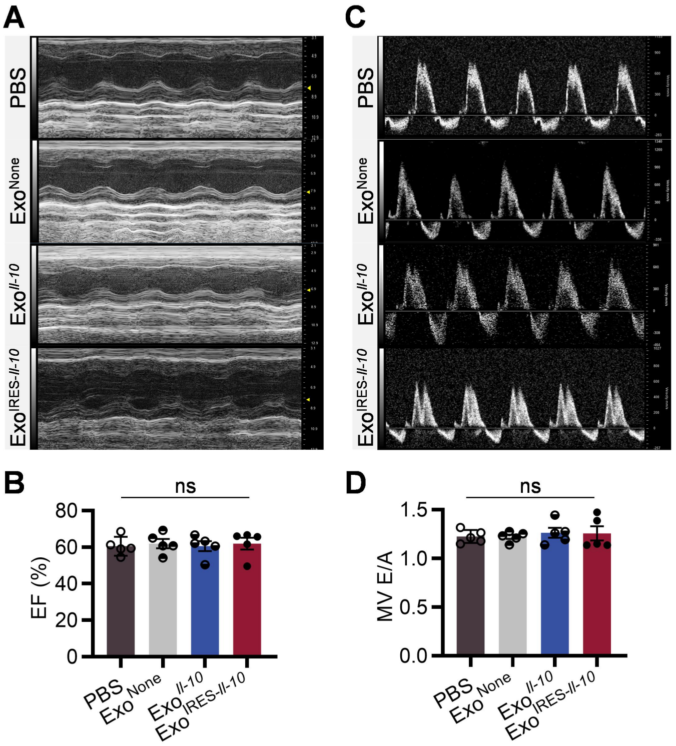

Figure S11 Cardiac function change in mice receiving different exosome treatments. (A) Representative images of M mode echocardiography of mice with indicated treatments. (B) Quantification of systolic function parameter EF. (C) Representative images of mitral flow Doppler echocardiography. (D) Quantification of diastolic function parameter E/A. Data are expressed as mean ± SD. Data are analyzed by one-way ANOVA. ns, no significant difference. n = 5 per group. EF, ejection fraction; MV, mitral valve.
